# Detection of Toxoplasma gondii from Clinical Specimens of Patients Receiving Renal Transplant Using ELISA and PCR

**DOI:** 10.5812/numonthly.13657

**Published:** 2013-11-13

**Authors:** Morteza Izadi, Nematollah Jonaidi Jafari, Abbas Mahmoodzadeh Poornaki, Javid Sadraei, Babak Rezavand, Hamid Reza Mirzaei, Hossein Zarrinfar, Abulfazl Khedive

**Affiliations:** 1 Health Research Center, Baqiyatallah University of Medical Sciences, Tehran, IR Iran; 2 Department of Parasitology, School of Medicine, Zanjan University of Medical Sciences, Zanjan, IR Iran; 3 Parasitology Department of Medical School, Tarbiat Modares University, Tehran, IR Iran; 4 Department of Immunology, School of Medicine, Tehran University of Medical Sciences, Tehran, IR Iran; 5 Department of Medical Parasitology and Mycology, Ghaem Hospital, School of Medicine, Mashhad University of Medical Sciences, Mashhad, IR Iran; 6 Bayer Paul (BP) Vaccine and Pharmaceutical Company, Tehran, IR Iran

**Keywords:** Toxoplasma, Kidney Transplantation, Enzyme-Linked Immunosorbent Assay, Polymerase Chain Reaction

## Abstract

**Background:**

*Toxoplasma gondii* is an opportunistic parasitic organism causing infection in many mammals, including immunosuppressed patients. Toxoplasmosis as an opportunistic infection is highly prevalent among patients receiving a kidney transplant.

**Objectives:**

The purpose of this study was to identify and determine the prevalence of *Toxoplasma gondii* in clinical samples collected from patients receiving renal transplants.

**Patients and Methods:**

A total of 50 blood samples and 40 lung lavage samples from transplanted patients admitted to the infectious wards and the patients undergoing bronchoscopy were collected. The B1 Gene of *Toxoplasma gondii* was amplified using PCR of the blood and bronoalveolar lavage BAL samples, and IgG and IgM antibodies against *Toxoplasma* were detected in serum samples using ELISA.

**Results:**

Our results indicated that anti-toxoplasma specific IgG and IgM antibodies were prevalent among transplant recipients with values of 54% and 4% respectively. PCR was performed to detect *Toxoplasma gondii* in 3 blood and lavage samples (3.3%) with 100% sensitivity and 97.9% specificity.

**Conclusions:**

*Toxoplasma gondii* pulmonary infection is measured along with brain toxoplasmosis in patients receiving a kidney transplant. After serological methods, PCR is the second useful method for *Toxoplasma gondii* screening. Proper prophylaxis before and after receiving a kidney transplant together with *Toxoplasma gondii* screening of donor and transplant is recommended.

## 1. Background

Toxoplasmosis is a zoonotic disease which infects all nucleated cells, but it cannot survive and multiply within red blood cells (1). for the first time in 1908, *Toxoplasma gondii* was detected by Nicolle and Manceaux in liver and spleen samples of the small rodent *Ctenodactylus gondii* (2). Felids are the definitive hosts of the microorganism, and most other mammals are known as the intermediate hosts. *Toxoplasma gondii* infection in humans can be divided into congenital or acquired infections (3). *Toxoplasma gondii* infection is seen in patients in two acute and chronic forms. When the immune system of human body is active, the cystic form of the parasite is observed (chronic form) but in the immune-compromised cases and patients with deficiency in immune system, active form of the parasite is presented, and then the clinical manifestations reveal (4). Life-threatening disease occurs in immunocompromised hosts such as AIDS patients, organ transplant recipients and patients with malignancies who are undergoing chemotherapy. The risk of acute toxoplasmosis among transplant patients who had not received anti-* Toxoplasma gondii* prophylaxis is remarkably high (5). The diagnosis of toxoplasmosis is mainly based on serological tests of disease-specific antibodies, imaging and molecular diagnosis using the clinical specimens (6).

Immunosuppressed patients infected with *Toxoplasma gondii* show symptoms such as diffuse encephalopathy, meningoencephalitis, extensive brain lesions and pneumonia (7). *Toxoplasma pneumonia* in immunosuppressed individuals caused55% of the mortalities (8). Patients received organ transplant administrate immunosuppressive drugs to prevent the rejection of the organ. Immunosuppression influences the patient resistance to a variety of opportunistic pathogens like *Toxoplasma gondii *(9). Early diagnosis of toxoplasmosis along with proper treatment can prevent serious consequences of infection (10).

## 2. Objectives

Due to the increasing number of patients receiving kidney transplants in our country, the aim of this study was to identify and determine the prevalence of *Toxoplasma gondii* among patients receiving a kidney transplant in Tehran.

## 3. Patients and Methods

This cross-sectional study was conducted on patients receiving kidney transplants. The samples were collected from patients admitted to specialized hospitals in Tehran due to various infections as well as the patients undergoing bronchoscopy. A total of 50 blood and 40 BAL samples were collected from specialized hospitals in Tehran to evaluate the titer of anti-*Toxoplasma gondii* antibody, 5 mL blood was taken from patients, and was divided into two equal portions in vials. Blood samples were moved to the laboratory immediately. For serological tests, serum was separated by centrifugation at1000rpmfor 5 minutes. The serums were kept at -20°C until the start of the experiments.

### 3.1. ELISA

ELISA for detection of anti-toxoplasma IgG and IgM antibodies was conducted using VIRO-IMMUN kit (Germany) according to the kit protocol. To measure the amount of IgG and IgM antibodies, 50 serum samples from renal transplant recipients were collected.

### 3.2. DNA Extraction

DNA was extracted from whole blood and BAL samples, using DNA extraction kit of MTB manufactured by Roche company, according to the manufacture protocol. The DNA extracted from whole blood samples and BAL was kept at-80°C until PCR was performed.

### 3.3. PCR

The specific primers TR1: (5´-ACGAACACTCGCAGAGATGA-3´) and TR2: (5´-GATCCTTTTGCACGGTTGTT-3´) was designed for B1 Gene. Deionized water was used as negative control, and RH strain (derived from an asymptomatic child with initials of R.H.) provided in Department of Parasitology of Tarbiat Modares University was used as positive control.

PCR was performedin a final volume of 25 µL by adding 0.8 µL of magnesium chloride, dNTP, Taq polymerase enzyme, 2.5 µL PCR buffer, DNA template and 1 µL of primers at a concentration of 1 pM.

Each of 35 cycles of PCR thermal was consisted of an initial denaturation cycle for 3 minutes at 94°C, denaturation of DNA for 30 seconds at 94°C, annealing for 30 seconds at 45°C, extension for 30 seconds at 72°C and final extension for 5 minutes at 72°C. 10 µL of the amplified PCR product was analyzed on 1.5% agarose gel electrophoresis and visualized under Transluminatorafter ethidium bromide staining.

### 3.4. Statistical Analysis

In order to measure the significance testing between age and IgG, IgM titers and PCR, independent T-test was conducted. To find the relationship between gender, duration of transplant and IgG and IgM titers and PCR, the fisher exact test was performed. All differences were considered significant at the level of P < 0.05. All statistical analysis was performed using the Statistical software for the social sciences (SPSS) version 20 (Chicago, Inc., USA).

## 4. Results

Of the 90 patients selected for study, 29 were female (32.2%) and 61 male (67.8%). The mean age was 36.62 ± 12.14 years. ELISA was applied to measure anti- *Toxoplasma *IgG and IgM antibodies. The results showed that among 50 blood samples of renal transplant patients, 27 patients (54%) were positive for IgG antibody and 2 patients (4%) were positive for IgM antibodies. *Toxoplasma gondii *-specific DNA amplification using PCR of blood and lung lavage samples collected from renal transplant patients revealed that 3 blood samples (6%) and 2 lung lavage samples (5%) were positive (Tables 1 and 2). 

**Table 1.** Diagnosis of *Toxoplasma gondii *in Blood Sample of Kidney Transplantations Patients Using ELISA and PCR

	Total	IgG Positive	P Value	IgM Positive	P Value	PCR Positive	P Value
**Duration of transplantation, No. (%)**			0.7		0.5		0.99
More than 6 months	21 (42)	12 (57.1)		0 (0)		1 (4.8)	
Less than 6 months	29 (58)	15 (51.7)		2 (6.9)		2 (6.9)	
**Age, Mean (SD), y**	35.54 (11)	37.74 (10.67)	0.13	33.50 (13.43)	0.79	30.66 (10.69)	0.44
**Gender, No. (%)**			0.001		0.99		0.57
Female	21 (42)	8 (27.6)		1 (4.8)		2 (9.5)	
Male	29 (58)	19 (90.5)		1 (3.4)		1 (3.4)	

**Table 2.** Molecular Diagnosis of *Toxoplasma gondii *in Lavage Sample of Kidney Transplantations Patients

	Count	PCR Positive, No. (%)	P Value
**Duration of Transplantation**			0.14
More than 6 months	25	0 (0)	
Less than 6 months	15	2 (13.3)	
**Age, Mean (SD), y**	37.97 (13.39)		0.23
**Gender, **			0.99
Female	8	0 (0)	
Male	32	2 (6.2)	

**Figure 1.**
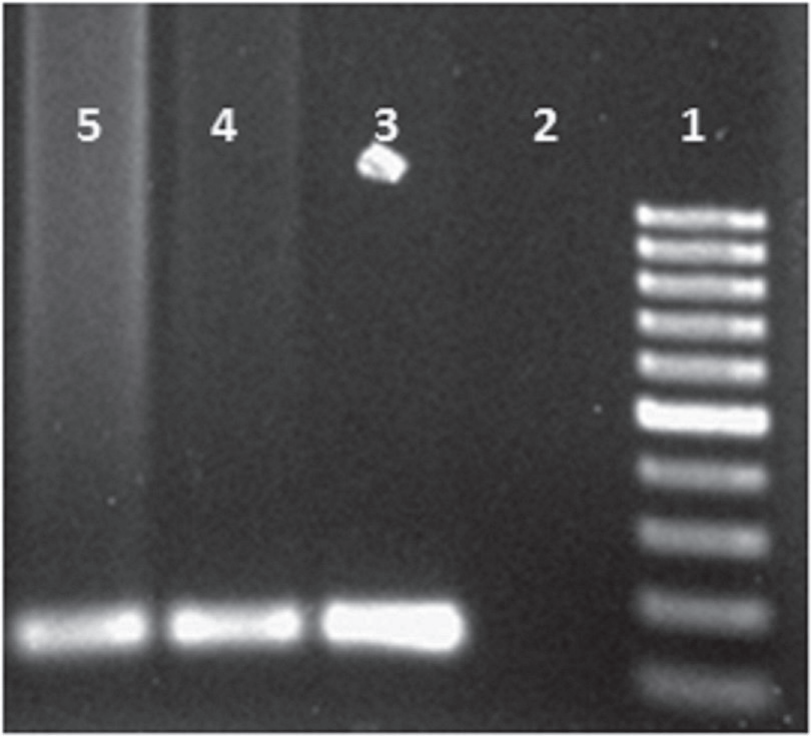
The First, Second, Third, Fourth and the Fifth Lanes Representing 100 bp Marker, Negative Control, Positive Control, Positive Blood Sample, and Positive Pulmonary Lavage Sample Respectively.

Positive samples were detected by presenting a 120 bp band of amplification test on 1.5% agarose gel (Figure 1). 

Sensitivity and specificity of PCR was measured according to the gold diagnosis standard of *Toxoplasma gondii *(i.e. ELISA). The studies showed that the sensitivity and specificity of PCR in detecting Toxoplasmosis was100% and 97.9%, respectively (Table 3). 

**Table 3.** Sensitivity and Specificity of PCR

PCR	ELISA IgG		Total	ELISA IgM		Total
	Negative	Positive		Negative	Positive	
**Negative**						
Count	22	25	47	47	0	47
% Within ELISA	95.7	92.6	94	97.9	0	94
**Positive**						
Count	1	2	3	1	2	3
% Within ELISA	4.3	7.4	6	2.1	100	6
**Total**						
Count	23	27	50	48	2	50
% Within ELISA	100	100	100	100	100	100

Statistical analysis showed no significant relationship between the age, duration of renal transplant in patients and IgG, IgM titers and PCR. There was no significant relationship between gender and IgM level and PCR. There was a significant correlation between positive IgG antibody level and female patients in this study.

## 5. Discussion

Immune system disorders in transplant recipients and cancer patients dispose these patients to infection of a variety of opportunistic pathogens due to immunosuppressive drugs intake(11). *Toxoplasma gondii* is considered as an opportunistic infectious microorganism among immunosuppressed patients. The first case of Toxoplasmosis in Iran was reported by Ansari et al. in 1948, which was isolated from conjunctiva of a 9-year old youngster (12). *Toxoplasma gondii* can cause multiple organ infection in immunosuppressed patients, such as lung secondary to CNS infection. Among patients with immune system disorders, patients with AIDS showed the highest prevalence of *Toxoplasma* pneumonia (13).

Many studies have reported a high incidence of cerebral and disseminated toxoplasmosis after receiving a kidney transplant. The anti-*toxoplasma gondii *prophylaxis can be directly related to decreased amount of toxoplasmosis. In patients receiving kidney transplants, the incidence of IgM antibody has been reported to be 24.1% within the first year of transplantation (14).

In this study, 50 blood samples, collected from transplant recipients, were assessed for IgG and IgM antibodies. Our results showed that the prevalence of anti-toxoplasma IgG and IgM antibodies were 54% and 4%, respectively. These results were in accordance with the results of Keshavarz (15). In our study, a significant correlation was found between positive IgG antibody level and female gender. There have been several reports of pulmonary toxoplasmosis inpatients receiving kidney, heart, bone marrow, liver, stem cell, pancreas and lung transplants (16-21).

*Toxoplasma gondii pneumonia* is diagnosed by different methods including serological tests, culturing and isolation of the microorganism. Derouin and colleagues in 1989 had detected the *Toxoplasma pneumonia* in three BAL samples and an autopsy specimen of patients with immune deficiency, by Methylene blue staining, indirect immunofluorescence and growing on MRC5 medium of (22).

Diagnosis of pulmonary toxoplasmosis in patients by molecular methods is important in immunosuppressed patients undergoing bronchoscopy due to the respiratory problems. The results have shown that the highest prevalence of pulmonary toxoplasmosis is reported among patients with AIDS, which has been up to 14% (23).

Molecular diagnosis of *Toxoplasma gondii* in our study was conducted on 40 lung lavage samples of renal transplant recipients. The results showed that 2 samples (5%) were positive for *Toxoplasma gondii*. Molecular diagnosis of toxoplasmosis among immunosuppressed non-AIDS patients showed a prevalence of 2 to 6.4% of pulmonary toxoplasmosis, which was consistent with our results (24, 25).

Chronic toxoplasmosis infection is directly associated with the use of immune-suppressive drugs and anti-*Toxoplasma gondii* prophylaxis. In patients receiving a kidney transplant who had negative *Toxoplasma* antibody before the transplantation, cessation of prophylaxis leaded to positive antibody against Toxoplasmosis (26, 27).

Detection of *Toxoplasma gondii* using PCR can be valuable in aligns with serological methods. PCR is very important method for appropriate diagnosis of pulmonary toxoplasmosis due to the high rate of mortality and morbidity rate of cases with toxoplasmosis (28). The screening of anti-*Toxoplasma gondii* antibodies in organ transplant patients without chronic infection is important once the donor with chronic *Toxoplasma* infection can transmit the infection to healthy transplant recipient (29). In this regard, screening of anti-*Toxoplasma gondii* antibodies in kidney donors and recipients is recommended before and after transplantation.
